# Autonomy and prevention: From conflicting to complementary aims of prenatal screening

**DOI:** 10.1111/bioe.13380

**Published:** 2024-11-10

**Authors:** Wybo Dondorp, Guido de Wert, Ellis C. Becking, Peter G. Scheffer, Mireille Bekker, Lidewij Henneman

**Affiliations:** ^1^ Department of Health, Ethics & Society Care & Public Health Research Institute (CAPHRI), Maastricht University Maastricht The Netherlands; ^2^ Department of Obstetrics Wilhelmina Children's Hospital, University Medical Centre Utrecht, Utrecht University Utrecht The Netherlands; ^3^ Department of Human Genetics Amsterdam Reproduction and Development Research Institute, Amsterdam University Medical Centre, Location Vrije Universiteit Amsterdam The Netherlands

**Keywords:** autonomy, ethics, Non‐Invasive Prenatal Test (NIPT), pregnancy complications, prenatal screening, prevention

## Abstract

From an ethical point of view, there is an important distinction between two types of prenatal screening. The first of these targets maternal or foetal conditions (e.g., infectious diseases, blood group sensitization) where early detection allows for interventions that improve the chances of a healthy pregnancy outcome. The second screens for foetal conditions such as Down syndrome, where a timely diagnosis in most cases only allows for a choice between preparation for a child with special needs or termination of the pregnancy. Whereas the former makes an easy fit with the prevention aim of most other population screening programmes, the latter does not. In order to steer clear from a possible eugenic reading of its aim, a wide international consensus has emerged for the view that prenatal screening of this type should have the atypical aim of helping women (couples) to make autonomous reproductive choices, rather than reducing the birth prevalence of the relevant disorders. However, keeping these types of prenatal screening apart may become increasingly difficult given the development of tests, such as the Non‐Invasive Prenatal Test, which cannot only be used for both types of screening but may also lead to interconnected findings on both sides of the divide. This makes it an urgent question: What the aim or aims of this new hybrid screening should be? As neither ‘prevention’ nor ‘autonomy’ will do, we argue for a normative framework that gives both aims their due, while recognizing the tensions between them.

## 1 INTRODUCTION

In most countries with prenatal screening programmes for chromosome abnormalities, their aim is typically defined in terms of providing pregnant women (or couples) with meaningful options for reproductive decision‐making. If a serious abnormality is found in the foetus, they may either prepare themselves for having a child with special needs or make a timely request for the pregnancy to be terminated. Recent developments in prenatal screening technology not only allow for an enlargement of the scope of such programmes in terms of the types and numbers of abnormalities that may thus be targeted but also affect the understanding of their aim. To the extent that findings more often include conditions that allow for timely preventative interventions, these programmes may come to be seen as serving a second aim: promoting healthy pregnancy outcomes, in addition to furthering reproductive autonomy. The normative implications of this merging of aims in future prenatal screening programmes are the focus of this paper.

## 2 TYPES AND AIMS OF PRENATAL SCREENING

We use the term ‘screening’ as it is used in public health policy discourse: a systematic offer of medical testing to a non‐patient population.^1^ As most screening programmes are aimed at reducing morbidity and/or mortality, the benefits for the target group are typically understood in terms of better health prospects as a result of timely treatment or prevention. Think here of neonatal screening, or different forms of cancer screening. Ideally, in these programmes, societal and individual benefits coincide where the screening succeeds in achieving its public health aim of reducing the burden of disease.

How would this apply to prenatal screening? Here, a distinction must be made between two main types of prenatal screening: screening for maternal or foetal conditions where early detection enables timely treatment or prevention and for foetal conditions where early detection in most cases only allows for the choice to ‘prepare or terminate.’^2^ The classical example of the former type is screening for infectious diseases in the woman, for example, HIV and hepatitis B, and blood group sensitization. We further refer to this as prenatal screening type 1 (PNS1). The classical example of the latter is prenatal screening for foetal Down syndrome, later expanded to common trisomies. We refer to this as prenatal screening type 2 (PNS2).

Whereas PNS1 makes an easy fit with the public‐health prevention aim of most other screening programmes, PNS2 does not. As it brings no health benefits for the woman or the child to be, it can only be brought under a prevention heading if seen as a tool for reducing the birth prevalence of the relevant disorders by giving pregnant women the option of terminating affected pregnancies. For instance, in the early days of prenatal screening for Down syndrome, the American epidemiologists Zena Stein and Mervyn Susser argued that it was desirable from a public health perspective to try to achieve an ‘almost total prevention of Down's syndrome (…) by screening all pregnant women’, adding that the success of this approach would depend on ‘the vigour with which the programme is explained (…) to the public.’^3^

### 2.1 Problems with reducing birth prevalence as prevention

Internationally, this ‘prevention account’ of the aim of PNS2 led to moral criticism. Apart from general concerns about the morality of abortion that for some remain a reason to categorically reject prenatal screening of this kind, the objections were twofold.^4^ First, there was a risk of women being subtly (or not so subtly) pressured to make the ‘right’ decisions, which was especially problematic in the light of the morally sensitive and highly personal nature of the ‘preparation or termination’ choices that this type of prenatal screening may present them with. Second, according to the so‐called ‘disability rights critique’, those living with the relevant conditions (or their representatives) would have reason to ask how welcome they really are in society.^5^

In a contribution to this debate, the British clinical geneticist Angus Clarke discerned two possible justifications for a prevention account such as advocated by Stein and Susser that he found both wanting, but for different reasons.^6^ The distinction is between prevention for cost‐reduction: sparing society the cost of (often) life‐long treatment of persons with the conditions screened for and prevention in the sense of avoiding serious suffering for persons who would otherwise be born with those conditions. As Clarke remarks, it is difficult to see how the former justification (avoiding expensive lives) would not amount to turning prenatal screening into a problematic form of eugenics. This is less obvious for a prevention account framed in terms of a societal obligation to avoid serious suffering. But here the problem is that this could only be maintained for screening with a rather limited scope. Clarke observes that for many conditions, such as Down syndrome, the suffering that the screening would help avoid ‘may not so much be of the future child, as of the parents and the family.’^7^

### 2.2 The qualified autonomy account

In the light of the problems arising with the prevention account, a broad consensus has emerged (at least in Western countries), stating that the aim of PNS2 should not be prevention, but rather enabling pregnant women or couples to make autonomous reproductive decisions.^8^ This alternative ‘autonomy account’ has also led to criticism by Clarke and others. For instance, would it not entail that contested reproductive choices such as foetal sex selection for non‐medical reasons should also be made available? As Clarke observes: although in all fields of health care (and public health), autonomous and well‐informed choices are important, ‘the simple maximization of such decisions is not a coherent goal.’^9^ Similarly, Stephan Wilkinson has argued against ‘pure reproductive autonomy’, which could never be the aim of a screening programme offered as a public health service and paid from public funds.^10^

While this is a valid point, we think it is a misunderstanding to read the ‘autonomy account’ of PNS2 as a call for promoting autonomy for its own sake. Clearly, this was not envisaged when the concept was put forward as an alternative for prevention in the debate about the aims of prenatal screening and subsequently enshrined in policy documents in many countries. Rather than suggesting that screening should be offered to enable pregnant women to make whatever reproductive choices they want, the idea was to enable them to determine for themselves whether the prospect of a child with possibly extensive medical needs is something they could accept or would rather choose to avoid. As the scope of the screening offer was (and still is) confined to serious abnormalities, this is not ‘pure’, but qualified autonomy.^11^

While this reading of the aim of PNS2 leads back to the notion of avoiding suffering, it differs from the view discussed by Clarke in two respects. First, it takes the possible suffering as referring not only to that of the child to be but also of the parents and the family. Second, as the aim is to give pregnant women or couples an autonomous choice in the matter, rather than to achieve any population‐level effects on the burden of suffering, the success of PNS2 programmes with this aim does not depend on maximizing the percentage of affected pregnancies that are terminated as a result. It is the choice, not the outcome that counts. Of course, this is not deny that offering PNS2 with this aim will de facto lead to a reduction of the birth of children with serious disorders, and therefore also to a reduction of suffering and lower healthcare costs.^12^

Note that this whole debate about ‘autonomy versus prevention’ is about the aim for which prenatal screening programmes are set up and systematically offered to pregnant women. On the level of individual women's motives for participating in the screening and deciding upon its findings, there is nothing wrong with saying that PNS2 enables them to opt for ‘prevention’ in the sense of a personal choice to avoid the birth of a child with a disorder or handicap. But precisely to ensure that this remains a personal choice rather than a societal norm, it is important to insist that PNS2 is aimed at serving reproductive autonomy rather than at achieving prevention.

### 2.3 Different aims: Implications for prenatal screening practice

Given that PNS1 serves the health interest of the woman and the future child, it is uncontentious to present this screening as a normal part of good quality prenatal care. Informed consent will of course be needed, but there is nothing seriously wrong with professionals advising women that having the screening is recommended. Nor is it problematic to evaluate PSN1 programmes in terms of their contribution to healthy pregnancy outcomes.

Things are different with regard to PSN2. Here, the message should be that having the screening is entirely optional: a personal choice that depends on one's values, ideals, and capacities. For professionals offering the screening or providing pre‐ or post‐test counselling, this means that they should as much as possible do so in a non‐directive way. Any form of pressure, however subtle, is at odds with the aim of the screening and undermines its justification. Moreover, evaluation should look at whether–or to what extent–the programme succeeds in providing pregnant women with meaningful options for reproductive choice, regardless of what it achieves in terms of health effects.

In the light of these opposite implications, it is important to avoid the two types of prenatal screening getting mixed up. Mixed messages about the aim of the screening offer might lead pregnant women or couples to assume that forms of PNS1 are not really recommended or that having PNS2 is to be seen as a matter of course.

## 3 EMERGING HYBRID SCREENING SCENARIOS

Avoiding such confusion may be quite a challenge for emerging scenarios in which both types of prenatal screening are combined in one and the same procedure. A current area of research is the potential for using present screening programmes for chromosome abnormalities with the Non‐Invasive Prenatal Test (NIPT) as a context to also screen for pregnancies at risk for complications related to poor placental function. For instance, the Dutch AFFIRM study assessed the predictive value of the foetal fraction (i.e. the amount of placental‐derived cell‐free DNA (cfDNA) in relation to the total amount of cfDNA in the blood of the pregnant woman), which is measured as a quality control parameter in NIPT. In this study, it was shown that a low foetal fraction in NIPT was associated with an increased risk of developing adverse pregnancy outcomes such as hypertensive disorders of pregnancy, small for gestational age neonates, spontaneous preterm birth, and diabetes.^13^ Another example of research exploring NIPT‐related markers of complication risks is a recent Belgian study indicating that cell‐free DNA methylation profiling in NIPT samples could be useful in first‐trimester prediction of preeclampsia.^14^

Follow‐up research is needed to establish that the predictive value of these markers is sufficiently robust and that this would enable interventions (monitoring, medication) that improve the odds of a healthy pregnancy outcome for mother and child. If so, this would lead to a potential new form of PNS1 that in itself might well fulfill the proportionality requirements of the normative framework for responsible screening. But the main ethical challenge lies with adding this to present NIPT screening for chromosome abnormalities. In addition to its current aim of providing pregnant women or couples with meaningful options for reproductive decision‐making, NIPT screening would then also have the–in itself non‐problematic–prevention aim of reducing perinatal morbidity and mortality. In other words, this would add a form of PNS1 to an existing PNS2 programme.

With the wide range of sequencing data accessible with NIPT, and explorations of how these could be used for preventative purposes, further hybrid screening scenarios are expected to arise. An example of this is a recent exploratory study on the potential benefits of adding a test for human cytomegalovirus (hCMV) to the cfDNA analysis already performed in NIPT screening for chromosomal abnormalities.^15^ The authors suggest that this approach has the potential for identifying women at risk of transmitting the virus to their foetuses, at a stage where this could still be avoided by the timely administration of therapeutic agents that are currently under investigation.

### 3.1 The case for separation

Already ten years ago, Jørgensen et al. have pointed out why hybrid screening might raise questions from a moral point of view.^16^ Their paper was in the form of an ethical commentary to a predecessor of current scenarios of hybrid screening. At the time, it was observed that combined levels of biomarkers forming part of, or considered for, the then‐standard first‐trimester testing approach used in prenatal screening for chromosome abnormalities, might also yield information about the woman's risk of developing preeclampsia. Those at a heightened preeclampsia risk could then be offered low‐dose aspirin as prophylactic medication. In their discussion of how this might lead to combined screening for both chromosome abnormalities and preeclampsia risk, Jørgensen et al. expressed a concern for ‘a desensitization’ for the special character of autonomy‐aimed screening: ‘(…) there are deep‐seated values at stake in the decisions to be made as a consequence of [prenatal screening for chromosome abnormalities], which are far less self‐evident than the ones made in pre‐eclampsia screening.’^17^ The authors feared that when both types of screening were offered in one package and performed in one procedure, both pregnant women and professionals might fail to realize this difference.

This concern was echoed in the qualitative research part of the AFFIRM study, investigating pregnant women's views on having screening for adverse pregnancy outcomes added to the present screening for chromosome abnormalities with NIPT.^18^ One participant in the focus group interviews spoke of a risk of ‘goal confusion’, meaning ‘that people will no longer be aware what the NIPT really is about.’ In several interviews, a recurring observation was also that making screening for pregnancy complications available as an add‐on was unfair to those who for whatever personal reasons would not have NIPT screening for chromosome abnormalities. While this reflects a more general justice problem with all forms of opportunistic screening that are only available for part of the wider target group, in this case the different aims add a further layer to that concern. As participants remarked, there is a risk of undue pressure on women to accept the full package including screening for chromosome abnormalities to have done everything that can be expected of a good future mother. In order not to constrain women's choices, it was recommended that using NIPT to predict adverse pregnancy outcomes should also be available for those who choose not to have screening for chromosome abnormalities.

While this would solve the specific concerns with respect to those intending to decline present NIPT screening, it would leave the more general concern about ‘goal confusion’ in place with respect to those still having both screenings in one procedure. This is why Jørgensen et al. called for a more radical separation. Their recommendation was to refrain from the efficiency benefits that are sought in combining forms of PNS1 and PNS2. Instead of making hybrid combinations, these forms of screening were to be offered in distinct procedures, so as ‘to be performed independently of one another.’^19^

### 3.2 Hybrid screening with interconnected findings

Jørgensen et al. do not specify what this would mean in practice. It would seem logical to think here of separate information and counselling sessions for forms of PNS1 and PNS2. And in the lab, what should be avoided is generating information that women have chosen not to be informed about. This, however, may not always be easy if the relevant findings are interconnected. For instance, in the AFFIRM‐scenario, it will not be a problem to determine the foetal fraction without also performing cfDNA analysis as a test for chromosome abnormalities. But the reverse is more challenging, given that the foetal fraction is used in most centres as a standard quality parameter for that test. Of course, this only leads to an information‐handling problem in cases where women have made an informed decision to decline screening for pregnancy complications, which can be expected to be much rarer than their declining screening for chromosome abnormalities, precisely indeed because of the different aims of these screenings. But such cases may still exist and would then cause a challenge for professionals, who of course would want to be informed about clinically relevant risk information that allows for timely prevention.

A more complicated scenario, in terms of interconnected findings, emerges with expanded NIPT, where this test is used to also screen for chromosome abnormalities beyond trisomies 21, 18, and 13. In the TRIDENT‐2 study, the use of genome‐wide NIPT led to finding structural chromosome abnormalities (SCAs: duplications and deletions of parts of chromosomes), as well as rare autosomal trisomies (RATs).^20^ The problem with RATs is that only in about 5% of findings, the abnormality is located in the foetus and will then lead to a serious disorder. In the rest of findings, RATs are only placental (confined placental mosaicism; CPM), and the foetus is not affected. However, these pregnancies are at increased risk of adverse outcomes such as preeclampsia, low birth weight, and preterm birth.^21^ The TRIDENT‐2 researchers therefore recommend tailored perinatal obstetric care in all pregnancies with CPM, including monitoring of placental function and foetal growth and medication.

Here, we see the contours of what may in the future become a further scenario of hybrid screening, where findings are interconnected in a way that renders separation impossible. The same test outcome either signifies a possibly serious abnormality in the foetus or a placental problem that may cause serious complications. As an invasive procedure is necessary to tell the difference, it will be needed to inform the woman about the finding at a stage where it is still unclear with which of these problems and related choices or options she may be confronted. In a small but significant minority, these will be typical PNS2 choices (whether or not to continue the pregnancy in the light of a serious foetal abnormality that does not allow for treatment or prevention). In most cases, by contrast, these will be the kind of preventative options typical of PNS1 (monitoring, medication).

It can be expected that more such forms of hybrid screening with interconnected findings will become available. With the further development of prenatal medicine, NIPT (or ultrasound) findings that now only give the woman a choice between ‘prepare or terminate’, may come to facilitate the further option of in utero treatment. One telling example is the possible future scenario of prenatal treatment (using FDA‐approved food supplements) aimed at improved postnatal cognitive functioning in children with Down syndrome.^22^ Should this become established treatment, this would give screening for Down syndrome a hybrid character that does not allow for separation, given that here again the same finding may either lead to reproductive decision‐making as in PNS2 or to a form of treatment as in PNS1, depending on the values and ideals of the woman (or the couple).^23^

### 3.3 Ultrasound screening as precedent

To see that separation of PNS1 and PNS2 may not always be possible, it is not necessary to only look at future scenarios. The existing practice of offering all pregnant women a first and second‐trimester anomaly scan is a clear precedent of interconnected hybrid screening. To the extent that ultrasound screening leads to findings with similar implications as current screening for chromosome abnormalities, it is a form of PNS2; to the extent that it reveals adverse conditions that can be remedied by a timely adaptation of pregnancy management or medication (or in presently still rare cases, surgical forms of foetal intervention), it should rather be labelled PNS1. But as it is impossible with ultrasound to only look for conditions that fall on one or the other side of this distinction, and as many findings fall on both, here again we have a form of hybrid screening that defies separation. It is at least remarkable that this has not until now been given much attention in the debate about the ethics of prenatal screening. However, from qualitative research amongst professionals offering routine ultrasound, it is clear that at least part of the felt challenges of this practice arise from tensions between its underlying autonomy (PNS2) and prevention (PNS1) aims.^24^

## 4 TOWARDS A NORMATIVE FRAMEWORK FOR HYBRID SCREENING

If it is indeed inevitable that in the future we will see more and more forms of hybrid screening that, due to interconnected findings, cannot be separated, then what should be the normative framework for guiding this practice?

### 4.1 Beneficence in utero: Why ‘just prevention’ will not do

One conceivable answer is that for interconnected forms of hybrid prenatal screening, we can simply do without the traditional autonomy aim of PNS2. After all, the reason for needing this atypical aim (as compared to all other forms of public health screening) was the fact that offering the screening would not contribute to prevention in the non‐problematic sense of improving health outcomes. But as for hybrid screening this is no longer the case, it would seem best to ‘revert to normal’ and to simply regard prevention as its aim.^25^

In what seems an instance of this line of thinking, Kevin Conley et al. define the aim of future comprehensive NIPT screening as ‘beneficence in utero.’^26^ As they say: ‘Beneficence for the future child, not autonomy of the parents, should be the guiding principle for any anticipated expansion of prenatal genetic testing.’^27^ We do not find this convincing. For even in the most optimistic scenario of scientific developments, there will be findings that leave no other option but the choice between terminating the pregnancy or preparing for a child with a serious disorder. Or it may be that available options for prenatal treatment are still experimental, or of limited effectiveness, or highly burdensome or risky for the pregnant woman. Think for instance of the complex choices that women (couples) may find themselves confronted with after a prenatal diagnosis of spina bifida. Should they accept and prepare, opt for a termination, or try to improve the postnatal health prospects of their future child by embarking upon risky foetal surgery?^28^

In its silence about the remaining reality of selective termination decisions, Conley et al.'s proposal raises the question how such decisions might be incorporated in their account. Should selective terminations be seen as a further form of beneficence towards the future child? Except in highly exceptional cases, this would be difficult to maintain.^29^ Or is the message that there should be no place for selective terminations in the new reality of beneficence‐aimed prenatal genetic screening? This would make ‘normalisation’ (back to prevention) a moral cause, very much in line with the agenda of the so‐called ‘pro‐life’ movement. From an ethical point of view, the problem with both readings is that by making beneficence for the future child the sole aim of prenatal genetic screening, chances are high that women's freedom to make their own reproductive decisions will be seriously curtailed.^30^

### 4.2 Prenatal personalized medicine: Why ‘just autonomy’ will do neither

The opposite conceivable answer is suggested in the following quote from Diana Bianchi. In a paper sketching a scenario in which hybrid screening would be part of a future field of prenatal personalized medicine,^31^ she remarks: ‘Historically, the goal of prenatal diagnosis has been to provide an informed choice to prospective parents. We are now at a point where that goal can and should be expanded to incorporate genetic, genomic and transcriptomic data to develop new approaches to fetal treatment.’^32^ We read this as saying that the autonomy paradigm should be broadened to cover a wider range of meaningful choices than only with regard to ‘termination or preparation.’ Following this lead, hybrid screening could be understood as aimed at helping women (or couples) make their own reproductive choices also with respect to treatment options that may improve the health outcomes of the pregnancy both for herself and the child to be. Clearly, this would avoid the problems of the ‘back to prevention’ proposal. However, it does so at the cost of replacing the one‐sidedness of that proposal by its own. To suggest that for hybrid screening we can simply stick to the autonomy aim of PNS2 is to ignore how its potential contribution to population health is a valid and important reason for making this screening (and subsequent forms of health care) available to pregnant women. Moreover, whereas counselling women for decision‐making in the context of PNS2 should be as non‐directive as possible, this need not always be the case where findings from hybrid screening are concerned. For instance, suppose that such findings lead to options for foetal treatment that would significantly improve the health prospects of the future child without exposing the woman to serious burdens or risks. And also that the woman has indicated she will not opt for a termination, but definitely wants to carry the pregnancy to term. Wouldn't it be acceptable in such cases for professionals, or even required of them, to counsel women to at least seriously consider having that treatment? The fact that this question cannot be dismissed out of hand clearly indicates that for hybrid screening programmes ‘just autonomy’ will not do.

### 4.3 Dealing with inevitable tensions

We argue that the hybrid character of emerging and future forms of prenatal screening is a challenge that cannot simply be overcome by bringing the relevant practices under the heading of either prevention or autonomy. A normative framework will therefore be needed that gives both aims their due, while recognizing the tension between them that will often be obvious. For instance, where, due to interconnected findings, screening for pregnancy complication risks will not be possible without revealing chromosomal abnormalities, autonomy considerations requiring professionals to be as non‐directive as possible when counselling women about participation in the screening, should prevail. Even though, in this scenario, professionals may find it desirable from a prevention perspective to have a high participation in the screening, this is not what should guide them in their counselling. The reason for this is that the woman's interests in making an autonomous decision with regard to screening that may lead to termination decisions has greater moral weight than the small chance that her non‐participation may negatively affect the outcome of the pregnancy for her child or herself. Clearly, the same high standard of non‐directiveness should guide counselling with regard to all further steps in the trajectory that may lead to a decision about whether or not to terminate the pregnancy, including that decision itself. However, as hybrid screening leads to a broader array of choices than only between preparation or termination, the health interests of the future child may loom so large that it would be morally problematic not to address them in counselling.

So what we need, in our view, is a new normative framework that acknowledges the hybrid character of many new forms of prenatal screening and tries to build norms for practice based on the relative importance of autonomy versus prevention considerations in specific constellations of information, choices, and risks.

## 5 CONCLUSIONS

We agree with Jørgensen et al. that combining forms of PNS1 and PNS2 in one hybrid screening programme should as much as possible be avoided. For instance, future NIPT‐based screening for adverse pregnancy outcomes or hCMV status should ideally be offered as part of a PNS1 programme, separate from NIPT‐screening for chromosomal abnormalities. This would reduce the risk of goal confusion and allow women to have the former screening while rejecting the latter. Clearly, this need not exclude that laboratory‐wise, testing can still be combined for women wanting information about both chromosomal abnormalities and adverse pregnancy outcomes or hCMV status.

However, where hybrid screening leads to what we have referred to as interconnected findings, such separation will not always be possible. Instead of trying to overcome the tension between the two aims of such screening, we need a framework that can do justice to both. Given that, as earlier indicated, ultrasound screening is an existing form of interconnected hybrid screening, the development of morally sound best practices may take its lead from experiences in that context.

## Footnotes

1 Juth, N., & Munthe, C. (2012). *The ethics of screening in health care and medicine: Serving society or serving the patient?* Springer. https://doi.org/10.1007/978-94-007-2045-9; Health Council of the Netherlands. (2008). *Screening: Between hope and hype*. Health Council of the Netherlands. https://www.healthcouncil.nl/documents/advisory-reports/2008/04/01/screening-between-hope-and-hype

2 Dondorp, W., De Wert, G., Bombard, Y., Bianchi, D. W., Bergmann, C., Borry, P., Chitty, L. S., Fellmann, F., Forzano, F., Hall, A., Henneman, L., Howard, H. C., Lucassen, A., Ormond, K., Peterlin, B., Radojkovic, D., Rogowski, W., Soller, M., Tibben, A., … Cornel, M. C. (2015). Non‐invasive prenatal testing for aneuploidy and beyond: Challenges of responsible innovation in prenatal screening. *European Journal of Human Genetics*, *23*(11), 1438–1450. https://doi.org/10.1038/ejhg.2015.57; De Wert, G. M. (2000). Erfelijkheidsonderzoek en ethiek: een gordiaanse knoop. *Wijsgerig Perspectief*, *40*, 150–156; De Jong, A., & De Wert, G. M. (2015). Prenatal screening: An ethical agenda for the near future. *Bioethics*, *29*(1), 46–55. https://doi.org/10.1111/bioe.12122

3 Stein, Z., Susser, M., & Guterman, A. V. (1973). Screening programme for prevention of Down's syndrome. *Lancet, 1*(7798), 305–310. https://doi.org/10.1016/s0140-6736(73)91551-1

4 Juth & Munthe, op. cit. note 1, pp. 33–42.

5 Parens, E., & Asch, A. (1999). The disability rights critique of prenatal genetic testing. Reflections and Recommendations. *Hastings Center Report, 29*(5), S1–22. https://doi.org/10.1002/mrdd.10056

6 Clarke, A. J. (1997). Prenatal screening. Paradigms and perspectives. In D. S. Harper & A. J. Clarke (Eds.), *Genetics, society and clinical practice*. (pp. 119–140). Bios Scientific Publishers.

7 Ibid: 123.

8 Dondorp, W., et al., op. cit. note 2; Health Council of the Netherlands, op. cit. note 1; Juth & Munthe, op. cit. note 1.

9 Clarke, op. cit. note 6, p. 123.

10 Wilkinson, S. (2015). Prenatal screening, reproductive choice, and public health. *Bioethics, 29*(1), 26–35. https://doi.org/10.1111/bioe.12121

11 De Wert, op. cit. note 2; De Jong & De Wert, op. cit. note 2.

12 John, S. (2015). Efficiency, responsibility and disability: Philosophical lessons from the savings argument for pre‐natal diagnosis. *Politics, Philosophy & Economics, 14*(1), 3–22. https://doi.org/10.1177/1470594x13505412

13 Becking, E. C., Scheffer, P. G., Henrichs, J., Bax, C. J., Crombag, N., Weiss, M. M., Macville, M. E., Opstal, D. V., Boon, E. M. J., Sistermans, E. A., Henneman, L., Schuit, E., & Bekker, M. N. (2023). Fetal fraction of cell‐free DNA in noninvasive prenatal testing and adverse pregnancy outcomes: A nationwide retrospective cohort study of 56,110 pregnant women. *American Journal of Obstetrics and Gynecology*, *231*(2), 244.e1–244.e18. https://doi.org/10.1016/j.ajog.2023.12.008

14 De Borre, M., Che, H., Yu, Q., Lannoo, L., De Ridder, K., Vancoillie, L., Dreesen, P., Ackervegan, M. V. D., Aerden, M., Galle, E., Breckpot, J., Keirsbilck, J. V., Gyselaers, W., Devriendt, K., Vermeesch, J. R., Calsteren, K. V., & Thienpont, B. (2023). Cell‐free DNA methylome analysis for early preeclampsia prediction. *Nature Medicine, 29*(9), 2206–2215. https://doi.org/10.1038/s41591-023-02510-5

15 Faas, B. H. W., Astuti, G., Melchers, W. J. G., Reuss, A., Gilissen, C., Macville, M. V. E., Ghesquiere, S. A. I., Houben, L. M. H., Srebniak, M. I., Geeven, G., Rahamat‐Langendoen, J. C., Sistermans, E. A., & Linthorst, J. (2024). Early detection of active Human CytomegaloVirus (hCMV) infection in pregnant women using data generated for noninvasive fetal aneuploidy testing. *EBioMedicine, 100*, 104983. https://doi.org/10.1016/j.ebiom.2024.104983

16 Jørgensen, J. M., Hedley, P. L., Gjerris, M., & Christiansen, M. (2014). Including ethical considerations in models for first‐trimester screening for pre‐eclampsia. *Reproductive Biomedicine Online, 28*(5), 638–643. https://doi.org/10.1016/j.rbmo.2014.01.013

17 Ibid: 642.

18 Becking, E. C., Henneman, L., Crombag, N. M. T. H., Bertorotta, J., Rijsbergen, Y., Bax, C. J., Vliet‐Lachotzki, E. V., Scheffer, P. G., & Bekker, M. N. (2024, June 14). Perspectives of pregnant women on broadening the scope of noninvasive prenatal testing from screening for foetal aneuploidies to prediction of adverse pregnancy outcomes: A qualitative study. *Prenatal Diagnosis*. *44*(9), 1033–1042. https://doi.org/10.1002/pd.6621

19 Jørgensen, J. M., et al., op. cit. note 16, p. 642.

20 Van Prooyen Schuurman, L., Sistermans, E. A., Van Opstal, D., Henneman, L., Bekker, M. N., Bax, C. J., Pieters, M. J., Bouman, K., Munnik, S. D., Hollander, N. S. D., Diderich, K. E. M., Fass, B. H. W., Feenstra, I., Go, A. T. J. I., Hoffer, M. J. V., Joosten, M., Komdeur, F. L., Lichtenbelt, K. D., Lombardi, M. P., & Dutch, N. C. (2022). Clinical impact of additional findings detected by genome‐wide non‐invasive prenatal testing: Follow‐up results of the TRIDENT‐2 study. *The American Journal of Human Genetics*, *109*(6), 1140–1152. https://doi.org/10.1016/j.ajhg.2022.04.018

21 Ibid.

22 Guedj, F., & Bianchi, D. W. (2013). Noninvasive prenatal testing creates an opportunity for antenatal treatment of Down syndrome. *Prenatal Diagnosis*, *33*(6), 614–618. https://doi.org/10.1002/pd.4134

23 De Wert, G., Dondorp, W., & Bianchi, D. W. (2017). Fetal therapy for Down syndrome: An ethical exploration. *Prenatal Diagnosis*, *37*(3), 222–228. https://doi.org/10.1002/pd.4995

24 Ahman, A., Edvardsson, K., Fagerli, T. A., Darj, E., Holmlund, S., Small, R., & Mogren, I. (2019). A much valued tool that also brings ethical dilemmas—A qualitative study of Norwegian midwives' experiences and views on the role of obstetric ultrasound. *BMC Pregnancy Childbirth, 19*(1), 33. https://doi.org/10.1186/s12884-019-2178-x; Edvardsson, K., Lalos, A., Ahman, A., Small, R., Graner, S., & Mogren, I. (2016). Increasing possibilities—Increasing dilemmas: A qualitative study of Swedish midwives' experiences of ultrasound use in pregnancy. *Midwifery, 42*, 46–53. https://doi.org/10.1016/j.midw.2016.09.009; Edvardsson, K., Mogren, I., Lalos, A., Persson, M., & Small, R. (2015). A routine tool with far‐reaching influence: Australian midwives' views on the use of ultrasound during pregnancy. *BMC Pregnancy Childbirth, 15*, 195. https://doi.org/10.1186/s12884-015-0632-y

25 Dondorp, W. & De Wert, G. (2017). The ‘Normalization’ of prenatal screening: Prevention as prenatal beneficence. In D. Schmitz, A. Clarke, & W. Dondorp (Eds.), *The fetus as a patient. A contested concept and its normative implications*. (pp. 144–153). Routledge (Taylor & Francis). https://doi.org/10.4324/9781315170749

26 Conley, W. K., McAdams, D. C., Donovan, G. K., & FitzGerald, K. T. (2017). Beneficence in Utero: A framework for restricted prenatal whole‐genome sequencing to respect and enhance the well‐being of children. *American Journal of Bioethics, 17*(1), 28–29. https://doi.org/10.1080/15265161.2016.1251655

27 Ibid: 28.

28 Ravindra, V. M., Aldave, G., Weiner, H. L., Lee, T., Belfort, M. A., Sanz‐Cortes, M., Espinoza, J., Shamshisaz, A. A., Nassr, A. A., & Whitehead, W. E. (2020). Prenatal counseling for myelomeningocele in the era of fetal surgery: A shared decision‐making approach. *Journal of Neurosurgery: Pediatrics*, 1–8. https://doi.org/10.3171/2019.12.PEDS19449

29 Clarkeburn, H. (2000). Parental duties and untreatable genetic conditions. *Journal of Medical Ethics*, *26*(5), 400–403. https://doi.org/10.1136/jme.26.5.400

30 Dondorp, W., & De Wert, G. (2017a). Ethical issues in maternal‐fetal medicine. In C. H. Rodeck & M. J. Whittle (Eds.), *Fetal Medicine: Basic science and clinical practice* (pp. 139–147). Elsevier.

31 Bianchi uses the term ‘fetal personalized medicine.’ Because the relevant choices may also regard the woman's own health, and because any ‘fetal’ health interests are better understood as those of the future child, we propose to speak of ‘prenatal personalized medicine.’

32 Bianchi, D. W. (2012). From prenatal genomic diagnosis to fetal personalized medicine: Progress and challenges. *Nature Medicine*, *18*(7), 1041–1051. https://doi.org/10.1038/nm.2829

